# Four major dietary patterns identified for a target-population of adults residing in Newfoundland and Labrador, Canada

**DOI:** 10.1186/s12889-015-1433-y

**Published:** 2015-01-31

**Authors:** Zhi Chen, Lin Liu, Barbara Roebothan, Ann Ryan, Jennifer Colbourne, Natasha Baker, Jing Yan, Peizhong Peter Wang

**Affiliations:** Division of Community Health and Humanities, Faculty of Medicine, Memorial University of Newfoundland, St. John’s, Newfoundland Canada; School of Public Health, Tianjin Medical University, Tianjin, China

**Keywords:** Dietary pattern, Factor analysis, Nutritional epidemiology

## Abstract

**Background:**

Very limited nutritional epidemiological studies conducted to explore the unique dietary exposure in Newfoundland and Labrador (NL). This study aims to identify and characterize major dietary patterns in the target-population from general adult NL residents and assess the associations with selected demographic factors.

**Methods:**

A total of 192 participants, aged 35–70 years, completed and returned a food-frequency questionnaire (FFQ) and participated in a telephone interview to collect demographic information. Dietary patterns were identified by common factor analysis. Univariate and multivariate linear regression analyses were used to assess determinants of the different food consumption patterns. Pearson’s correlation coefficients were calculated for food scores of each pattern, total energy, and energy-adjusted nutrient intakes.

**Results:**

Factor analyses identified four dietary patterns, which were labeled as “Meat”, “Vegetable/fruit”, “Fish”, and “Grain” patterns. In combination, the four dietary patterns explained 63% of the variance in dietary habits of the study population. Multivariate linear regression analysis indicated an increasing trend of factor scores for Meat and Grain pattern with age. Male participants were found to be more likely to choose the Meat and Fish patterns. Current smokers and those married/living together tend to choose the Grain pattern. Pearson’s correlation coefficients showed positive correlations between fat and cholesterol and the Meat pattern, fiber and the Vegetable/fruits pattern, protein and the Fish pattern, and carbohydrates and the Grain pattern.

**Conclusion:**

This study derived four dietary patterns and obtained their significant associations with specific demographic characteristics in this population. It identified one dietary consumption pattern (Fish) not yet seen in other studied populations. These findings will update the current dietary-health information published in this province, and contribute to further research into the association between dietary practices and health.

## Background

Traditional approaches to nutritional epidemiology have focused on the associations of diseases with one or a small number of specific nutrients or foods [1,2]. Given that people eat a variety of foods with a complex combination of nutrients, the single-nutrient approach may fail to take into consideration the complicated interaction among nutrients, the potential confounding by an individual’s eating pattern, and the statistically significant associations by chance [3]. In order to overcome these limitations, an increasing number of researchers have begun to use food consumption patterns to characterize a population’s dietary intake and to examine potential relationships of these patterns with health [4-7]. Such an analysis of dietary patterns may provide a more accurate and comprehensive description of actual dietary exposure. Several studies have identified the modern “Western” dietary pattern, characterized by high intake of meat, highly processed foods, and sweets [2,8-11]. In contrast, a healthier pattern referred to as “Healthy or Prudent”, is characterized by higher intake of fruits, vegetables, legumes, whole grains, poultry, and fish [11]. These two major dietary patterns are not only associated with health outcomes, but have also been shown to be related to age, gender, living area, educational attainment, and other baseline demographic characteristics. For example, findings of Park SY et al. [12] and also Schulze MB et al. [13], suggest that in Hawaii, Los Angeles and some European populations that older residents are more likely to practice the vegetable-based dietary intake pattern over the Western pattern. Also, a study conducted in the US has suggested that, urbanites tend to choose the healthier dietary pattern as compared to rural residents and women have a healthier dietary pattern than men [14].

It is widely believed that dietary and cultural differences exist between Newfoundland and Labrador (NL) and the rest of Canada due partly to geographic isolation [15]. The life expectancy is lower [16] and the rates of death due to such chronic illnesses as cardiovascular disease and diabetes mellitus are higher than in any of the other ten Canadian provinces [17,18]. However, very limited nutritional epidemiological research has been conducted to examine unique characteristics of the NL diet. Additionally, because the 2004 Canadian Community Health Survey (CCHS Cycle 2.2, Nutrition Focus) [19] did not contain some foods commonly found in the NL diet, such as, pickled meat and cloudberry (bake-apples), it may not have portrayed an accurate representation of this population’s dietary intake. It could be that dietary intakes of this population were not well estimated by analysis of CCHS data. Therefore, there is a need to investigate the dietary patterns of NL residents, in order to know more about the true current food consumption patterns of this population and to see if such dietary patterns can provide insight into the elevated rates of illness experienced in the province.

Realizing the potential value of studying this particular population with its somewhat unique dietary characteristics and the higher rates of certain illnesses, our research group has recently developed and validated a food-frequency questionnaire (FFQ) for use with this population [20]. The objectives of this study are to proceed with the next step of a larger investigation of this population by using this tool to make a preliminary evaluation of the dietary patterns in one subgroup of the NL population, the adult, and to assess whether these patterns vary according to demographic characteristics.

## Methods

Dietary data used in this study were collected in the Canadian province of NL between February 2011 and May 2012.

### Sampling design and sample size

According to the 2011 Census Information and Statistics [21], the population of NL is approximately 514,536, with over 57% rural residents. A stratified random digit dialing [22] with proportional allocation sampling methodology was adopted for this study. Geographically, the survey covered the whole of NL, including both the urban and rural areas.

With the intention of measuring food intake for the general adult population of NL, the following inclusion criteria were used. An eligible participant was required to be:

1. A non-institutionalized adult resident of NL who had lived in NL for at least two years at the time of the study;
2. 35–70 years of age;
3. Able to speak and read English at the 8th grade level; and
4. Without the following conditions at the time of the study: cognitive impairment, psychological challenges, or pregnancy.

Therefore, using a list of landline telephone numbers provided by Info Canada, an initial random sample of 450 participants from the general population was recruited by telephone. A total of 306 persons were identified as eligible respondents and were sent the survey packages. Two hundred five (205) individuals participated in the survey, giving a response rate of 67.0%. This research was approved by the Health Research Ethics Board (HREB) [23] at Memorial University.

### Dietary intake assessment method

A self-administered food-frequency questionnaire (FFQ) was used to collect food consumption information among the NL adult population. The FFQ was modified from the Hawaii FFQ to account for the unique food consumption habits in NL. The original Hawaii FFQ was designed to assess the typical food intake of individual males and females in a multi-ethnic Hawaiian/Southern Californian population [24-27]. In the adapted NL FFQ, food items considered unusual in NL (for example, tamales and ham hocks) were deleted or altered while some items commonly consumed in NL (for example, moose meat and pickled meat) were added. The NL FFQ consists of 169 food items and includes a number of composite dishes that may contain multiple ingredients [20]. The foods listed in the FFQ are categorized into nine major groups: (1) beverages (other than liquid milk), (2) dairy products, (3) mixed dishes, (4) vegetables, (5) meat and fish, (6) cereals and grains, (7) fruits, (8) desserts and sweets, and (9) miscellaneous.

Participants were required to recall the frequency with which they usually consumed each item, choosing only one from the following options provided for each food/beverage item: (1) serving per day, (2) serving per week, (3) serving per month, or (4) rarely or never. In addition, subjects were requested to indicate the number of servings habitually consumed at a single sitting. An “average” portion, a standard serving expressed in household measures or grams, was provided for each food item or beverage in the FFQ. Respondents who consumed an amount different than the “average” portion provided were given the option of choosing “smaller” or “larger” portion sizes. A smaller size was defined as a portion approximately 75% or less of the average portion size, while a larger one was approximately 125% or more of the average size.

If a food item was consumed on a seasonal basis, the respondent was not only asked to estimate the frequency of the food item consumed during its season, expressed as times per day/week/month, or never/rarely, but also to indicate the length of the particular food’s season (for example, consuming cloudberry 2 times per week for 3 months only).

Demographic information--age, gender, size of community, marital status, employment status, level of education, and smoking habits--was collected by telephone interview. The current study involved the secondary analysis of data collected for FFQ validation. Thus, certain potential confounding factors of interest were not available to us.

### Data analysis

According to the nutritional characteristics and the usual frequency of consumption in this population, the 169 food items in the FFQ were grouped into 39 predefined categories based on the role of each food in the diet. Several foods (for example, eggs, beer, jam, and pies) comprised their own groups since they were considered inappropriate for combination. Nutrient intakes for individuals were calculated using the Elizabeth Stewart Hands and Associations (ESHA) Food Processor database software [28], and were adjusted for total energy intake with the use of the residual method [29] to obtain factors uncorrelated with total energy intake. If a participant reported consuming food that was not present in the database, the most appropriate alternative was chosen through a discussion with the research team or by consultation with academic nutrition experts.

Exploratory factor analysis of the reported number of servings of the various food groups was used to define the food consumption patterns within this population. The terms ‘Principal component analysis’ and ‘exploratory factor analysis’ are used interchangeably in much of the literature. To be consistent with our previous work, only the term—‘exploratory factor analysis’ was used in this study. Bartlett’s Test of Sphericity (BTS) and the Kaiser-Meyer-Olkin (KMO) measurement of sample adequacy were used to verify the appropriateness of factor analysis. Exploratory factor analysis was used for factor extraction. Factors were also orthogonally rotated (Varimax option) to achieve simpler structure with greater interpretability. Factors were retained based on the following criteria: factor eigenvalue > 1.35, identification of a break point in the scree plot, the proportion of variance explained, and factor interpretability [30]. The strength and direction of the associations between the patterns and food groups were described through a rotated factor loading matrix. Items were considered to load on a factor if they had a factor loading >0.5 [31]. Each individual received a factor score calculated for his/her dietary pattern to indicate the extent to which the diet corresponded to that pattern.

Univariate analyses and multivariable linear regression models were used to assess the associations between participants’ dietary patterns and demographic variables, with factor scores of each dietary pattern being the dependent variable. Because four dietary patterns were derived for this sample, four linear regression models were fitted to explore the associations. Those demographic factors were coded and entered into linear regression models as independent variables. Details are as following: age in years (1: 35–40, 2: 41–50, 3: 51–60, 4: 61–70), gender (1: female, 2: male), size of the participant’s community (1: less than 10,000, rural area; 2: more than 10,000, urban area), education attainment (1: some school but no high school certificate, 2: high school certificate, 3: post-secondary education), marital status (1: single, 2: separated/divorced, 3: widowed, 4: married/living together), and current smoker (1: yes, 2: no).

Pearson’s correlation coefficients were calculated between the factor scores of each pattern and energy-adjusted nutrient intakes so that the correlation between dietary patterns and specific nutrient intakes could be studied. Statistical analyses were performed using the Statistical Analysis System (SAS, version 9.2) software and the Statistical Package for Social Science (SPSS, version 10.5). Differences with *p*-value <0.05 were considered to be statistically significant.

### Ethical consideration

This research was approved by the HREB at Memorial University of Newfoundland. (Reference number 14.098).

## Results

### Demographic information

Out of a total of 205 questionnaires received by June 2012, we excluded participants who had left over 20 continuous items blank on the FFQ (n = 5) and those who reported energy intakes outside the range of 500–5000 kcal (n = 8). The latter exclusion matches the exclusionary rules for food-frequency questionnaire data used by Willett [29]. The remaining 192 respondents were involved in all further analyses. Comparison of selected demographic characteristics between respondents and non-respondents were made, with the only significant difference being the age profile of responders (35 to 40 years, 9%; 41 to 50 years, 24%; 51 to 60 years, 41%; 61 to 70 years, 26%) and non-responders (35 to 40 years, 16%; 41 to 50 years, 34%; 51 to 60 years, 32%; 61 to 70 years, 18%), p = 0.0032. Based on these differences we conducted a separate factor analysis with respondent data weighted to the age profile of the NL population. Results demonstrated little meaningful difference between weighted and un-weighted analysis.

Table 1 presents the social and demographic characteristics of the study sample. The sample consisted of 43 men and 149 women, aged 35 to 70 years, with a mean age of 55.0 ± 8.7 years. Most participants were non-smokers (82.8%) and had completed post-secondary education (59.4%). When stratified by gender, no significant differences in demographic characteristics were found between groups (data not shown).

**Table 1:** **Demographic characteristics of the study participants from Newfoundland and Labrador general adult population (n = 192)**

**Characteristics**	**n (%)**
Age range (years)	
35-40	15 (7.8%)
41-50	42 (21.9%)
51-60	77 (40.1%)
61-70	58 (30.2%)
Gender	
Males	43 (22.4%)
Females	149 (77.6%)
Living Area	
Rural area	111 (57.8%)
Urban area	81 (42.2%)
Education attainment	
Some school but no high school certificate	27 (14.0%)
High school certificate	51 (26.6%)
Post-secondary education	114 (59.4%)
Marital status	
Single	15 (7.8%)
Separated/divorced	18 (9.4%)
Widowed	8 (4.2%)
Married/living together	151 (78.6%)
Current Employment	
Part-time	16 (8.3%)
Full-time	74 (38.5%)
Seasonal	15 (7.8%)
Not employed	84 (43.8%)
Retired	61 (31.8%)
Not retired	21(11.0%)
No answer provided	2(1%)
Unusable data	3 (1.6%)
Currently smoking daily	
Smoker	33 (17.2%)
No	159 (82.8%)
Previous smoking daily	
Yes	84 (43.8%)
No	75 (39.0%)
N/A	33 (17.2%)

### Factor analysis

**Figure 1:**
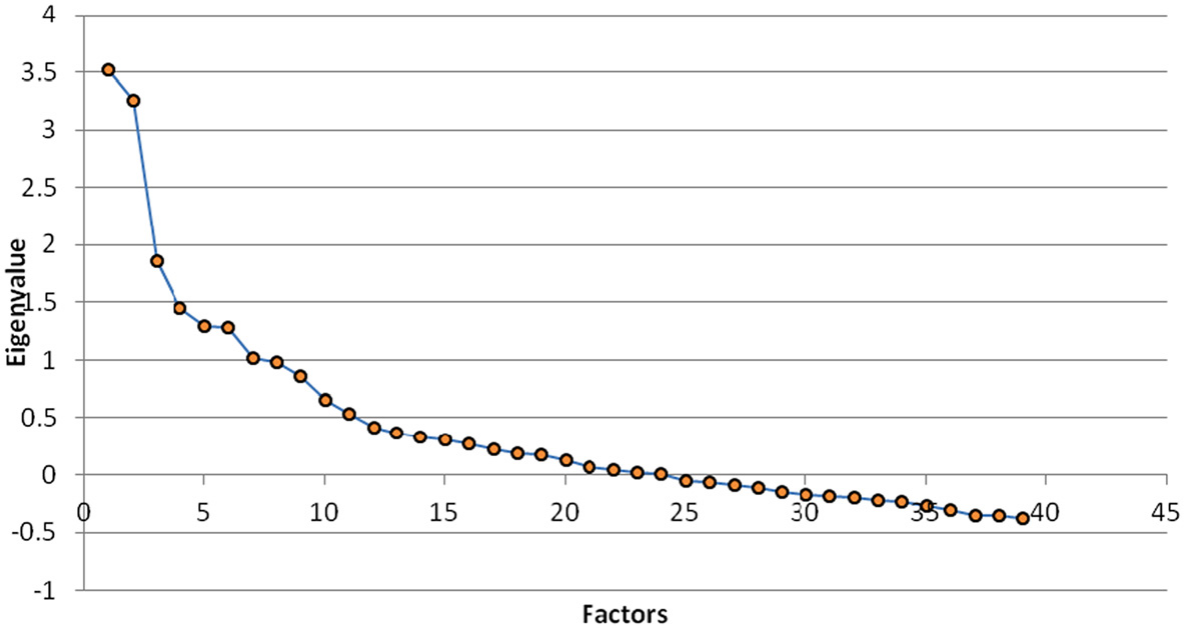
**Scree plot test in factor extraction (common factor analysis).**

The observed KMO was 0.602 and therefore the sample was considered to be adequate for factor analysis. The BTS was significant (p < 0.001), indicating homogeneity of variance by the food consumed. Figure 1 shows the scree plot of eigenvalues for each factor. The first four eigenvalues, which were 3.53, 3.25, 1.85, and 1.44 respectively, dropped substantially. After the fifth factor (1.29), the values remained more consistent (1.28 for the sixth and 1.02 for the seventh factor). As a result, a 4-factor solution was selected. These four factors accounted for 63% of the variability of food consumption within the sample. Some studies have found that factor solutions differ by gender [1,32]. Therefore, we conducted factor analyses separately for men and women. We found no difference in the number of food consumption patterns between genders (data not shown).

The four retained factors were identified as four dietary patterns and were labelled Meat, Vegetable/fruit, Fish and Grain, according to the results obtained from the factor loading matrix (Table 2), where a higher factor loading of a given food group indicates a greater contribution of that food group to the specific pattern. We named the first pattern Meat, since it is characterized by a high consumption of red meat, cured/processed meat, and cured/processed red meat. Conversely, the Vegetable/fruit pattern has an emphasis on several vegetable/fruit groups, including greens, tomato sauce, berries, and other vegetables. The Fish pattern indicates a preference for fish and processed fish. The final pattern was labeled as Grain because of the high positive loadings in whole grains, cereals, and grains, and negative loadings in the groups containing beer, white wine, and coffee.

**Table 2:** **Factor Loadings and Explained Variances (VAR) for the Four Major Dietary Patterns identified in an adult NL population, using a common factor analysis** ^**a**^

**Food groups**	**Factor loading**			
	**Meat**	**Vegetable/fruit**	**Fish**	**Grain**
Milk	−0.16		0.18	0.43
Yogurt		0.30		0.32
Coffee		0.18		−0.31
Tea			0.17	0.19
Sugar				
Soft drinks	0.42		−0.15	−0.20
Egg	0.16			
Cheese		0.25		
Mixed dishes	0.32			
Red meat	**0.83**			
Game			0.21	
Cured/processed red meat	**0.90**			
Cured/processed meat	**0.93**		0.20	
Poultry	0.21	0.32		
Fish	0.22	0.16	**0.78**	
Processed fish	0.31		**0.70**	
Fruit juice		−0.25		0.38
Other fruits		0.34		0.30
Root vegetables		0.31	0.37	
Cruciferous vegetables		0.33	0.22	
Other greens		**0.68**		
Beans, peas		0.29	0.45	
Tomato sauce	0.16	**0.60**		
Other vegetables		**0.75**	0.23	
Total cereals and grains	0.16	0.17		**0.55**
Whole grains		0.30		**0.52**
Desserts and sweets	0.22			0.21
Vegetable juice			0.34	0.26
Beer				−0.24
White wine				−0.26
Red wine	−0.18	0.26		
Liquor				
Citrus		0.20	0.17	0.20
Berries	−0.15	**0.50**		
Dried fruit		0.39		
Canned fruit	0.16			0.34
Pies, tarts				
Jam, jelly				0.30
Pickled vegetables				017
Proportion of VAR explained (%)	22%	20%	12%	9%
Cumulative VAR explained (%)	22%	42%	54%	63%

^a^Absolute values less than 0.15 were not listed and those above 0.50 indicated in bold to visually emphasize strength of association.

### Linear regression analysis

The results from the multivariate regression analysis shown in Table 3 indicated that the overall models, which included all demographic information, were significant for the Meat (F = 3.28), Fish (F = 2.42), and Grain (F = 6.81) patterns, while the model fitted for Vegetable/fruit pattern (F = 2.10) is not significant. Older people are more likely to choose Grain pattern but less likely to have a Meat pattern. Male participants are more likely to exhibit the Meat and Fish patterns. Current smokers and those married/living together prefer the Grain pattern. The rest of the demographic factors were not related to the scores for any pattern.

**Table 3:** **Association between various pattern scores and selected demographic characteristics in this study population as assessed by multivariate linear regression analysis**

	**Meat** ^**ǂ**^	**Vegetable/fruit**	**Fish** ^**ǂ**^	**Grain** ^**ǂ**^
Age	−0.15*	0.10	0.15	0.16*
Gender	0.18*	−0.12	0.18*	−0.12
Living area	−0.13	0.06	0.05	−0.07
Education attainment	−0.05	0.16	−0.11	−0.07
Marital status	−0.06	0.02	0.02	−0.33*
Currently smoking daily	−0.11	0.11	−0.01	0.22*

*β are significant at p < 0.05.

^ǂ^indicates significant multivariate model (p < 0.05) included all the demographic information.

The association of the factor scores for each dietary pattern with total energy and energy-adjusted nutrient intakes are illustrated in Table 4. Scores of the Meat pattern have positive significant association with total energy, fat, sodium, cholesterol, and calcium intakes, as well as significant negative associations with carbohydrate and fibre intakes. The Vegetable/fruit pattern scores were positively correlated with total energy, fiber, and sodium. With the Fish pattern, the higher the factor scores, the higher the protein intake and the lower the fat intake. The grain pattern was characterized by high intakes of total energy, carbohydrates, and calcium, but with low intakes of sodium, fat, and cholesterol. Pearson’s correlation coefficients between factor scores of each dietary pattern and absolute nutrient intakes were also calculated. According to the results, correlations between factor scores of each dietary pattern and absolute nutrient intakes are similar in magnitude to those between factor scores and energy-adjusted nutrient intakes.

**Table 4:** **Pearson’s correlation coefficients of dietary pattern scores with total energy and energy-adjusted nutrient intakes**

	**Meat**	**Vegetable/fruit**	**Fish**	**Grain**
Energy	0.39**	0.38**	0.28**	0.55**
Protein (g)	0.12	0.12	0.31**	0.09
Carbohydrate (g)	−0.26**	−0.10	0.10	0.40**
Fiber (g)	−0.29**	0.59**	0.23**	0.10
Fat (g)	0.18*	0.05	−0.24**	−0.32**
Na (mg)	0.36**	0.22**	0.27**	−0.10
Cholesterol (mg)	0.22**	−0.05	0.11	−0.05
Calcium (mg)	−0.36**	0.04	0.03	0.32**

Correlation is significant at *p < 0.05 and **p < 0.01.

## Discussion

Although the NL diet is known to be unique and is suspected to play an important role in the high incidence for several diseases, there have been no studies that systematically assess NL dietary patterns. Results from the present study added new knowledge that contributes to future nutritional epidemiological research. We identified four major dietary patterns, Meat, Vegetable/fruit, Fish, and Grain, from a sample of the adult population of NL. The total variance explained by the four aforementioned food patterns was 63%, with the largest variance, 22%, being explained by the Meat pattern. After fitting two linear regression models to explore the associations between factor scores of dietary patterns and demographic factors, no main effect of the demographic factors on the Meat pattern was found. Associations between education attainment and Vegetable/fruit, gender and fish, age/marital status and Grain pattern were found.

The Meat pattern, with a high consumption of red meat, processed/cured meat, and processed/cured red meat, is similar to the set of food items referred to as the Western pattern in many previous studies [33,34]. This pattern has been reported to have associations with adverse outcomes such as cancer [35], cardiovascular diseases [14,36], and obesity [2]. The second pattern identified in the current study, Vegetable/fruit, is comparable to the Prudent and Vegetable/fruit patterns described in other studies [12,33,37]. This pattern consists mainly of vegetables, tomato sauce, and fruits. Studies describe this pattern as the most desirable or healthy diet for a population, since it has been shown to be associated with a decreased risk of coronary heart disease [38], type 2 diabetes [33], colorectal cancer [39], and mortality for all groups who follow this dietary pattern. The Fish pattern, characterized by high consumption of fish and processed fish, seems to be unique to the NL population and is unlike any pattern described in other research. This phenomenon may be attributed to geographic isolation and the historical importance of the cod fishery in NL [40]. The final pattern, Grains, shares common elements with the “cereals” or “cereal-based” patterns discussed in several previous publications [13,41].

According to the results of linear regression analysis, the factor scores were associated with several demographic factors, including age, sex, marital status and current smoking status. Consistent with previous studies [12,13], age was found to have a negative relationship with the Western diet and a positive association with vegetable-based patterns. Older respondents in this study were less likely to follow the Meat pattern and more likely to follow the Fish pattern. However, no significant effect of age on the Vegetable/fruit pattern was observed. Previous studies have reported that women and urban residents tend to have higher loadings on healthy dietary patterns [12-14]. Our results showed that women are likely to have lower scores for the Fish patterns. Moreover, our findings indicated that living in urban or rural areas and attaining a high level of formal education are not associated with individuals’ dietary patterns. This is inconsistent with Park’s [12] results, which suggest that individuals with higher scores for a healthy dietary pattern tend to be more educated than those scoring lower. The results from our study pertaining to marital status support a hypothesis that dietary patterns may be influenced by marital status [42]. Those who self-reported as being married and/or living together were more likely to choose the Grain pattern than those who were single and/or divorced, or widowed. No significant correlation was found between marital status and other food patterns. Finally, current daily smoking daily was positively associated with the Grain pattern in our study. This contrasts with the results of some other studies [12,13].

Dietary pattern analysis has been criticized by some due to predefined food groups and self-labeling factors based on an investigator’s own interpretation of the data. The present study attempted to further characterize such factors and explain the labeling by calculating the correlation of the patterns’ scores with total energy and energy-adjusted nutrient intakes. Similar to the results of the majority of studies which have investigated dietary patterns, the Meat pattern (similar to the Western pattern proposed in other studies) was associated with higher energy, fat, cholesterol, and sodium, as well as lower carbohydrate and fiber. Our Vegetable/fruit pattern was very similar to the Prudent pattern described in other research and correlated with high fiber intake [12,33,37,43].

There are some limitations to the present study. The use of factor analysis requires some arbitrary decision-making regarding the assignment of foods to food groups, the number of retained factors, the method of rotation, and the labels of components [44]. While factor analysis using predefined food groups is commonly used in nutritional epidemiological research [1,2,45], it is potentially useful to compare differences when using predefined food groups versus the raw food items. As part of a sensitivity analysis, we also conducted factor analysis based on the 169 original food items in the FFQ, which only explains 16% of total variation. Thus, we believe the predefined food group approach is both more practically meaningful and statistically advantageous. Secondly, the FFQ, although a useful tool to measure dietary exposures, requires participants to recall their past dietary habits, often one or two years prior to the investigation. Consequently recall bias and social desirability bias are unavoidable. Thirdly, while aids were provided, participants were asked to self-report their eating habits. Information bias may have resulted especially when estimate of quantities of foods consumed are considered. Potential selection bias may exist because people who agree to participate in diet-health study are more likely to have an interest in healthy lifestyles and to practice healthier eating behaviours. As for any cross-sectional study, the researchers do not know how well findings, in this case dietary patterns, reflect population bahaviours of the past or future. Additionally, this study was based on secondary data analyses and so we were constrained from exploring the association between some potentially important demographic factors and factor scores, such as obesity. Use of secondary data also means that the researchers did not conduct sample size calculations, participants’ recruitment, and power analysis for this study.

The fast growth of mobile phone only users in the past two decades poses a great challenge to the traditional random-digital-dialing recruitment approach. Because our study participants were recruited through land-line phones, mobile phone only users would have been missed. According to Statistics Canada, 56% of all Canadian households used landline phones in 2013 [46]. Phone use is strongly patterned by age. Among households with members under 35 years of age, the percentage using cell phones only is much lower than among those households with members aged over 55 (60.6% vs. 6.4%) [46]. Given the study participants were aged 35 to 70 years, it might therefore be expected that the lower proportion of younger participants compared to the NL population might be due to both non-response in this age group as well as patterns of phone ownership.

Although we were faced with challenges and our study may not be powered to address the study objectives, it has several strengths. First, our subjects belongs to an understudied group with unique experiences/characteristics which when studied could potentially contribute to the understanding of that important association between dietary intakes and health status. Not only did we have access to this group of respondents but we had access to a tool developed specifically for use with the NL adult population and this tool, an FFQ, has been pretested to have a moderate measure of relative validity. In addition, few studies have considered gender differences as they pertain to food consumption patterns. We conducted factor analyses stratified for different genders, though no significant difference was found. Plus, as significant difference in age groups was found between respondents and non-respondents, to further estimate and adjust the effect of age, we conducted sample weights and weighted factor analysis based on 2011 census data in NL. Results suggest that there is no considerable difference between weighted and non-weighted analysis. Finally, we not only labelled the retained four factors but also explained the correlations between specific nutrient intakes and factor scores behind the labels that are emphasized by Slattery [47].

This study is an initial attempt to utilize our newly developed FFQ with a population subgroup at a higher risk of ill health in many regards as compared to other Canadian adults. This preliminary investigation has identified food patterns which characterize the consumption pattern of adult residents of NL. Future research is required to verify that these patterns truly represent the larger population of the province. Comparison of these dietary patterns with those practiced in other regions of the country could be informative. Further investigations into the unique Fish pattern identified by this study could also prove to be valuable.

## Conclusion

In conclusion, the present study provides an initial investigation into the dietary patterns practically adult residents of NL, a subgroup of the Canadian population with comparatively high rates of such diseases as cardiovascular disease and diabetes mellitus. We identified four major food consumption patterns in this population: Meat, Vegetable/fruit, Grain, and Fish, the latter of which has not yet been identified in studies of dietary intake patterns in other geographic areas.

## Abbreviations

NL: Newfoundland and Labrador
FFQ: Food-frequency Questionnaire
CCHS: Canadian Community Health Survey
ICEHR: Interdisciplinary Committee on Ethics in Human Research
ESHA: Elizabeth Stewart Hands and Associations
BTS: Bartlett’s Test of Sphericity
KMO: Kaiser-Meyer-Olkin
SAS: Statistical Analysis System
SPSS: Statistical Package for Social Science
